# Design and Validity of Randomized Controlled Dental Restorative Trials

**DOI:** 10.3390/ma9050372

**Published:** 2016-05-13

**Authors:** Gerd Göstemeyer, Uwe Blunck, Sebastian Paris, Falk Schwendicke

**Affiliations:** Department of Operative and Preventive Dentistry, Charité—Universitätsmedizin Berlin, 14197 Berlin, Germany; gerd.goestemeyer@charite.de (G.G.); uwe.blunck@charite.de (U.B.); sebastian.paris@charite.de (S.P.)

**Keywords:** clinical trial, dental restorations, evidence-based dentistry, randomized controlled trial, risk of bias, trial registration

## Abstract

*Background*: The evidence stemming from trials on restorative materials is shaped not only by trial findings, but also trial design and validity. We aimed to evaluate both aspects in randomized controlled dental restorative trials published from 2005–2015. *Methods*: Using systematic review methodology, we retrieved trials comparing restorative or adhesive dental materials. Two authors independently assessed design, risk of bias, registration status, and findings of trials. Descriptive and regression analyses were performed. *Results*: 114 studies on 15,321 restorations placed mainly in permanent teeth of 5232 patients were included. Per trial, the median number of patients was 37 (25th/75th percentiles: 30/51). Follow-up was 24 (20/48) months. Seventeen percent of trials reported on sample size calculations, 2% had been registered. Most trials (90%) used US Public Health Service (USPHS) criteria, and had a high risk of bias. More recent trials were more likely to have been registered, to have reported on sample size calculations, to be of low risk of bias, and to use other than USPHS-criteria. Twenty-three percent of trials yielded significant differences between groups. The likelihood of such differences was significantly increased in older studies, studies with potential reporting bias, published in journals with high impact factor (>2), longer follow-up periods, and not using USPHS-criteria. *Conclusions*: The majority of dental restorative trials published from 2005–2015 had limited validity. Risk of bias decreased in more recent trials. Future trials should aim for high validity, be registered, and use defined and appropriate sample sizes, follow-up periods, and outcome measures.

## 1. Introduction

While randomized controlled trials remain the cornerstone of clinical research, there is increasing focus on the validity of clinical studies. Biased trials have been found to potentially over-estimate the efficacy of the test *versus* control group [1,2]. Following a study’s rationale and hypothesis, trials should have sufficient power to detect worthwhile differences at a level of statistical significance. The quality and comprehensiveness of reporting was found to impact on the credibility and applicability of trials [3], which is one reason why registration of clinical trials is increasingly seen as a requirement for performing such trials, thus reducing risks of selective reporting. Design aspects, like unit of randomization or choice of outcome measure, might impact on trial findings. Publication bias might distort the truly-found comparative effects, as significant findings are more often published [4]. Last, all these parameters are likely to change with time given the increasing public or academic demand for a more effective and efficient use of research resources [5].

One of the most prolific fields in dental research is restorative material science, as restorations remain the most frequently-performed dental treatments [6,7,8]. While not all mentioned aspects are always possible to address in restorative trials, studies should nevertheless strive for high external and internal validity: previous studies involving other dental research disciplines have highlighted significant shortcomings of dental randomized controlled trials [9]. It is unknown if restorative trials are of different validity, or use different designs. Moreover, possible changes in trial design and validity over time have not been assessed. We investigated the quality and design of trials on dental restorative materials, and evaluated their development within the last decade to identify areas with improving quality and those where trials remain prone for bias, decreasing the strength of evidence. Our review question was: in randomized controlled trials on humans in need of restorative dental treatment, comparing one restorative material against another, what is the validity of these trials, as reflected by trial design, risk of bias, and reporting?

## 2. Materials and Methods

### 2.1. Study Design

We assessed the quality (risk of bias, appropriateness of statistical methods, registration) and design (unit of randomization, outcome measure) of dental restorative trials (*i.e.*, those investigating restorative or adhesive dental materials) based on a systematic review. The present evaluation is part of several analyses on restorative dental trials and their design (comparator choice and industry bias), as well as findings (network meta-analysis of the risk of failure).

### 2.2. Eligibility Criteria

Randomized controlled clinical trials (RCT) comparing the survival of two or more different restorative and/or adhesive materials in primary or permanent teeth of human subjects were included. RCTs needed to have been published between 2005 and 2015, as our focus was the assessment of quality and design of recent trials. RCTs were excluded if they compared different treatment techniques (e.g., conventional *versus* atraumatic restorative treatment), not materials, or placed restorative materials or adhesives as a sealant or for orthodontic bonding, not a restoration. 

### 2.3. Information Sources, Search, and Selection

**Figure 1:**
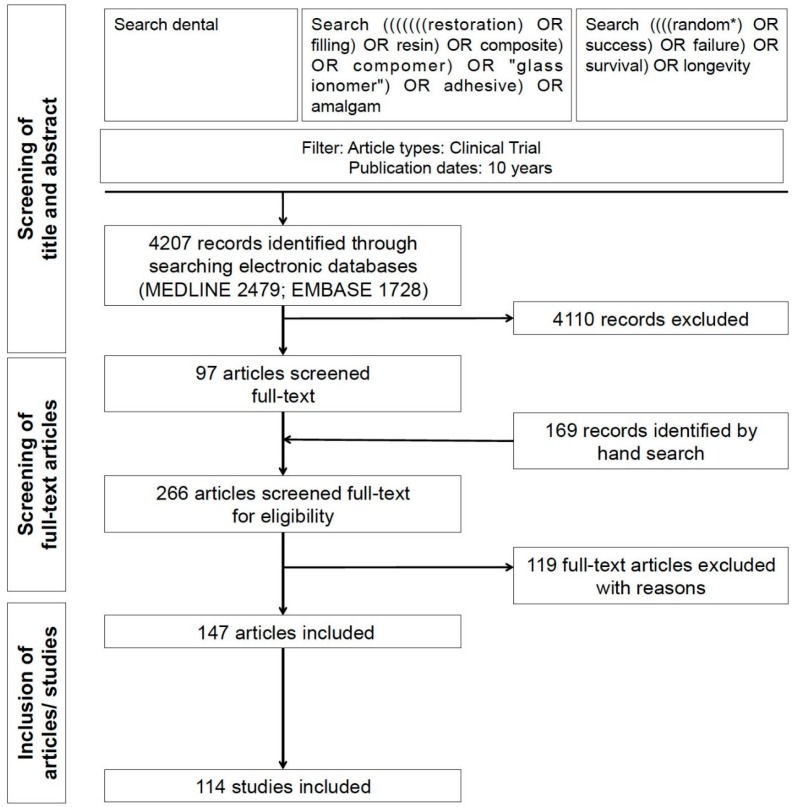
Flowchart of the search.

The Cochrane Central Register of Controlled Trials, MEDLINE (via PubMed) and EMBASE (via OVID) were searched on 2 March 2015 for relevant publications. The search strategy and screening procedure are shown in Figure 1. Further articles were identified by cross-referencing from retrieved full-text studies. Two calibrated reviewers (GG, FS) independently screened titles and abstracts of the identified studies for eligibility. Inclusion of studies was independently decided by both reviewers. Consensus was obtained by discussion.

### 2.4. Data Collection Process and Items

Duplicative data extraction was performed independently by two reviewers (GG, FS) using a piloted spreadsheet. If data of one clinical trial was published at different follow-up times, only data from the most recent publication (longest follow-up) were extracted. Data were recorded according to guidelines outlined by the Cochrane Collaboration [10]. The following data were extracted, but not all were used in the present analysis:

- Restorative and adhesive material: class, name, manufacturer.
- Trial design and methods: setting (primary or secondary care), number of patients; number of teeth; number of lesions; follow-up and drop-out, outcome measure (USPHS; Federation Dentaire International (FDI); Hickel; Vanherle; other), trial registration, unit of randomization (single teeth/patients, tooth pairs, *i.e.*, splitmouth, clusters of teeth or patients), method of analysis (analysis of participants regardless of whether they received the intervention or were available for follow-up, or analysis of participants based on the intervention they received and their availability for follow-up).
- Included teeth: dentition (primary or permanent), cavity location (cervical, *i.e.*, Black class V), load-bearing (*i.e.*, class I/II), or non-cervical and non-load-bearing (*i.e.*, class III/IV), indication for treatment (caries/replacement or non-caries lesions).
- Failures: number of failures per group, statistically significant different failure rates between groups. Failure was defined as restorations being lost or requiring restorative re-intervention (due to secondary caries, fracture *etc.*).
- Risk of bias: as recommended by the Cochrane Risk of Bias tool [1], the domains of sequence generation, allocation concealment, operator blinding, examiner blinding, attrition bias and selective reporting were used. Note that for operator and examiner blinding, we assessed both reported blinding and possibility of blinding, *i.e.*, blinding was not assumed when materials were clearly distinguishable when placing and evaluating them, even if studies reported on blinding.

In case of disagreement between reviewers, consensus was achieved by discussion. Inter-reviewer agreement was calculated for risk of bias and was found perfect (κ = 0.94).

### 2.5. Analysis

The unit of statistical analysis was trials. Descriptive statistics were employed following data distribution, assessed via the Shapiro-Wilk test. Comparisons between groups were performed using Chi-square or the Mann-Whitney U test, with Bonferroni correction to account for alpha inflation by multiple testing. To assess which factors were associated with trials finding statistically significant differences between groups, multivariable logistic regression analysis was performed, with variables being first entered simultaneously, and variables which did not significantly contribute to the model (*p* > 0.1) being removed stepwise (backwards). To assess how different trial properties changed over time within the last decade, bivariate linear or logistic regression was performed.

## 3. Results

### 3.1. Search and Studies

Searching electronic databases revealed 6641 articles. After screening titles and abstracts, 97 articles were assessed full-text. 169 further articles were identified by screening the reference lists of the 97 retrieved articles and existing systematic reviews. Eventually, 114 studies (147 articles) were included (Table S1), while 119 articles were excluded (Figure 1). Risk of bias of included studies can be found in Table S2.

### 3.2. Trial Properties

Within the included studies, a total of 15,321 restorations had been placed in 5232 patients. The median (25th/75th percentiles) number of included patients and lesions was 37 (30/51) and 105 (83/144), respectively, indicating high clustering (Table 1). After a median follow-up period of 24 (20/48) months, 91% of restorations remained for re-examination.

In 104 (91%) studies, restorations were placed in the permanent dentition, while 9 (8%) studies assessed restorations in primary teeth; one study did not report on the dentition. Trials were performed mainly in secondary care settings. 57 (50%) and 53 (46%) trials investigated restoration of cervical and load-bearing cavities, respectively. Four trials assessed non-cervical, non-load-bearing cavities.

Only few trials stated a hypothesis or reported on sample size calculations. From 114 trials, 2 (2%) reported on having been registered a priori. Most trials used USPHS criteria for outcome assessment, and nearly all trials did not sufficiently report on allocation concealment or blinding of operators. Only 26 (23%) trials found significant differences of failure rates between groups.

### 3.3. Changes of Trial Properties with Time

More recent trials tended to include more lesions and be registered more often, but this trend remained statistically non-significant (Table 2). More recent trials reported on sample size calculations significantly more often (OR [95% CI]: 1.35 [1.10/1.65]), and used randomization of pairs of teeth (splitmouth design) and USPHS criteria less often. Risk of selection, performance and detection bias was also significantly reduced in more recent trials.

### 3.4. Factors Associated with Significant Differences

The chance of trials finding significant differences between groups was reduced in more recent trials (Table 3). Studies published in journals with high impact (IF > 2) found significant differences significantly more often than those published in lower-impact journals (OR [95% CI]: 3.26 [1.25/10.8]). The same was found for trials with longer follow-up and those not using USPHS criteria. Trials in load-bearing cavities had significantly reduced chances of detecting significant differences between groups. Trials with a high risk of selective reporting found significant differences 12 times as often compared with trials without this risk.

## 4. Discussion

This study evaluated the design and validity of restorative dental trials published in the decade 2005 to 2015. Such evaluation is relevant, as it aids to assess the strength of the totality of evidence in the field and to identify improvements or areas with remaining weaknesses.

We found most dental restorative trials at high risk of bias, mainly in the domains of allocation concealment (93%) and performance (99%) or detection bias (46%). While operator blinding and blinding of examiners might be difficult or, when comparing different material classes, even impossible, allocation concealment is performable for all trials. The relevance of such concealment has been shown in various fields [11,12,13,14,15]. The impact of blinding examiners might be less relevant for “hard” outcomes like failure, as these are less prone for interpretation and subsequent detection bias [11]. The low proportion of trials with sufficient allocation and blinding processes is in line with findings from implant dentistry, where only 22% of trials reported or used proper concealment methods [11]; similar findings have been reported from both other medical and dental disciplines [9,16,17]. It was reassuring that in all of these domains of bias, improvement seems under way, with more recent trials being significantly more often blinded and concealed. It should be noted that we did not assess blinding of participants, which might be similarly difficult if materials can be distinguished visually, but might also be less important for an outcome like failure, which is usually not dependent on subjective interpretation by patients.

We found risk of selective reporting to be limited, with only three trials falling into this domain. Within these studies, selective reporting was detected by comparing the outcomes listed in the methods section of the articles with those eventually reported. However, detection of selective reporting might have been more accurate if study protocols had been available from trial registration for all assessed studies. In fact only two trials had been registered *a priori*. Dental journals should generally demand and enforce prospective registration [18,19], as this might not only reduce the risk of selective reporting and publication bias, but also improve the quality and design of trials by laying early focus on aspects which impact on validity later on. Our findings are in line with those reported for trials in implant dentistry [11], but in contrast to a much higher registration rate (approximately 45%) of general medical trials [20,21]. The latter might be due to different legal requirements for drugs than dental material trials.

Trials on restorative dental materials had sample sizes between 8 and 456 patients (median 37), *i.e.*, were relatively small. These trials only seldomly found significant differences between materials. This non-significance might be due two reasons: first, materials might really not be different, *i.e.*, show no clinically worthwhile difference in failure rates *etc*. To prove such non-difference, the performed trials needed to employ non-inferiority designs, a notion which our analysis does not support given that none of the trials mentioned such design or used an appropriate sample size calculation. Second and more likely, the lack of significant differences might be due to a lack of power, *i.e.*, type II error [22]. In line with this assumption, most studies did only inadequately describe sample size calculations, which is worrisome given that under- or overpowered trials are increasingly seen as a waste of resources and unethical [5]. In our study, 17% of trials had reported on sample size calculation (while only 5% formulated a hypothesis, which could be seen as prerequisite for defining the determining variables). This number is similarly low as that from implant dentistry (12%) and other medical and dental fields [22,23], as is the detected trend that newer trials increasingly use and report their considerations on sample size and relevant differences between groups. It should be noted that reporting of sample size calculation seems different depending on the journal of publication, and seems significantly higher in high-impact journals [9].

The median follow-up of trials was two years, and 91% of lesions were retained for analysis in the median after that time. Given that dental restorations fail long-term, with biological failures (mainly caries adjacent to restoration margins) occurring late during the lifetime of restorations [24], such follow-up time might be inadequate. Moreover, event rates remain low in this short-term period, which again impacts on the studies’ power, as demonstrated by the association between follow-up times and the chance of detecting significant differences between groups. For trials with longer follow-up periods, handling of potential attrition bias needs to be considered, especially if clustered or parallel-group designs had been used. More than 81% of the included trials did not account for a possibly imbalanced attrition (18% did not have any lost patients and did not need to consider such analyses). Given that analyses accounting for attrition tend to decrease statistical power even further, even fewer studies might have yielded significant results if such analyses had been applied. Future trial designs should consider the unit of randomization and the handling of drop-outs to achieve sufficient power to detect worthwhile differences between groups.

Most trials included in this analysis used USPHS criteria to assess restoration quality. The outcome we used for analysis of significant differences between groups, failure rate, can be derived from these criteria. USPHS criteria have been shown to have limited sensitivity, and several domains and categories of these criteria might not fully reflect on clinical success of restorations [25,26]. That and the advent of internationally-agreed new criteria (e.g., FDI or preceding systems) led to a decreased use of USPHS criteria recently. Trials which employed other than USPHS criteria detected significantly different failure rates more than four times than those employing USPHS criteria. It should be noted that this might not only be due to higher sensitivity of these criteria, but a great number of other factors, one being that not all new systems are fully validated.

In line with other dental [4,27] and medical disciplines [28,29,30], we found trials yielding significant differences more often being published in high impact journals. It might be that these trials are more rigorously designed (which was not confirmed by our analysis) or had greater power (which we did not check, but given that sample size was included as an independent variable within the model, is very unlikely). However, it should be considered that this association reflects publication bias, with significant findings being perceived as more relevant or having a greater impact [31].

## 5. Conclusions

Investigated dental restorative trials had limited validity, sample sizes, and follow-up periods. The risk of bias decreased in more recent trials. Future trials should aim for high internal validity, be registered *a priori*, and use defined and appropriate sample sizes, follow-up periods, and outcome measures. Reporting should follow the CONSORT criteria [3].

## Figures and Tables

**Table 1:** Properties of randomized controlled trials on dental restorative materials published 2005–2015.

Variable	Median (25/75th Percentiles) or Number (Percentages)
Patients per trial	37 (30/51; range: 8–456)
Lesions per patient	3.0 (2.1/4.0; range: 1.0–9.0)
Number of lesions per trial	105 (83/144; range: 36–1108)
Follow-up per trial	24 (20/48; range: 6–156)
Percentage of lesions retained at follow-up	91 (77/99; range: 53–100)
Trials with load-bearing/cervical/other cavities	53 (47%)/57 (50%)/4 (3%)
Trials in permanent/primary dentition/not reported	104 (91%)/9 (8%)/1 (1%)
Trials in primary/secondary care	3 (3%)/111 (97%)
Trials published in journals with impact factor >2	30 (26%)
Trials which stated a hypothesis	6 (5%)
Trials which described sample size calculation	19 (17%)
Trials which randomized a tooth/a tooth pair/a cluster	82 (72%)/30 (26%)/2 (2%)
Trials which performed intention-to-treat analysis/did not require such analysis as no attrition	1 (1%)/20 (18%)
Registered trials	2 (2%)
Trials using outcome measure other than USPHS	11 (10%)
Trials with unclear/high risk of bias	112 (98%)
Trials with unclear/high risk of sequence generation	70 (61%)
Trials with unclear/high risk of allocation concealment	106 (93%)
Trials with unclear/high risk of operator or participant blinding	113 (99%)
Trials with unclear/high risk of examiner blinding	52 (46%)
Trials with unclear/high risk of missing data	19 (17%)
Trials with unclear/high risk of selective	3 (3%)
Trials yielding significant differences between groups	26 (23%)

Median (25th/75th percentiles) or number of trials (percentages from overall trials) are reported.

**Table 2:** Development of trial properties with time.

Continuous Variables	β (95% CI)
Number of patients	0.19 (−0.98/4.44)
Lesions per patient	0.05 (−0.05/0.13)
Number of lesions	8.70 (−1.53/18.9)
Follow-up time (months)	1.40 (−0.48/3.19)
Lesions retained at follow-up	0.00 (−0.01/0.01)
**Binary variables**	**OR (95% CI)**
Trials on load-bearing cavities (ref.: cervical)	1.03 (0.91/1.17)
Trials in permanent dentition (ref.: primary)	1.22 (0.96/1.55)
Trials in secondary care (ref.: primary care)	0.91 (0.61/1.35)
Trials which stated a hypothesis (ref.: not stated)	1.27 (0.92/1.77)
Trials which described a sample size calculation (ref.: not described)	**1.35 (1.10/1.65)**
Trials which randomized tooth pairs (ref.: randomized teeth)	**0.90 (0.76/1.00)**
Registered trials (ref.: not registered)	**1.91 (0.69/5.32)**
Trials using outcome measure other than USPHS (ref.: USPHS)	**1.31 (1.02/1.69)**
Trials with sequence generation unclear/high (ref.: low)	**0.77 (0.67/0.89)**
Trials with allocation concealment unclear/high (ref.: low)	**0.49 (0.28/0.86)**
Trials with blinding of operators unclear/high (ref.: low)	**0.35 (0.04/2.96)**
Trials with blinding of examiners unclear/high (ref.: low)	**0.83 (0.73/0.94)**
Trials with missing data unclear/high (ref.: low)	1.03 (0.87/1.21)
Trials with selective reporting unclear/high (ref.: low)	0.91 (0.62/1.33)
Trials yielding significant differences between groups (ref.: no significant differences)	0.89 (0.76/1.03)
Intention-to-treat analysis performed/not required (ref.: required, but not performed)	0.97 (0.83/1.14)

Regression coefficients (β) or odds ratios (OR) with 95% confidence intervals (95% CI) were used to describe changes of properties with time (*i.e.*, per year). Positive regression coefficients or OR > 1 indicate a positive association, while negative regression coefficients and OR < 1 indicate a negative association. Statistically significant results and are highlighted in bold.

**Table 3:** Factors associated with studies yielding significantly different failure rates between groups.

Variable	Model 1 (Simulttaneously)	Model 2 (Backwards)
Fit	*R*^2^ = 0.31, *p* < 0.001	*R*^2^ = 0.19, *p* < 0.001
	OR (95% CI)	OR (95% CI)
Year of publication of trial	0.78 (0.62/0.99)	**0.81 (0.68/0.96)**
Journal impact >2 (ref.: ≤2)	3.53 (0.72/17.3)	**3.26 (1.25/10.8)**
Number of patients	1.02 (0.98/1.07)	
Lesions per patient	0.66 (0.26/1.71)	
Follow-up time (months)	1.01 (0.98/1.04)	**1.02 (1.00/1.03)**
Retained lesions (%)		
Trials with load-bearing cavities (ref.: cervical)	0.29 (0.12/7.32)	**0.29 (0.09/0.94)**
Trials in permanent dentition (ref.: primary care)	3.3 (0.04/3.00)	
Trials describing sample size calculation (ref.: not described)	2.48 (0.26/23.7)	
Registered trials (ref.: not registered)	n/a	
Trials using outcome measure other than USPHS (ref.: USPHS)	7.46 (8.69/64.0)	**4.77 (1.07/21.3)**
Trials with sequence generation unclear/high (ref.: low)	3.61 (0.78/16.8)	
Trials with allocation concealment unclear/high (ref.: low)	n/a	
Trial with blinding of operators unclear/high (ref.: low)	n/a	
Trials with blinding of examiners unclear/high (ref.: low)	0.51 (0.14/1.87)	
Trials with missing data unclear/high (ref.: low)	0.44 (0.05/3.92)	
Trials with selective reporting unclear/high (ref.: low)	557 (5.6/55,442)	12.1 (0.90/123)
Trials accounting for attrition in analysis/trails without attrition a (ref.: attrition not accounted for)	1.75 (0.64/4.78)	

For regression analyses, variables were either entered simultaneously (Model 1), or entered and then removed backwards (Model 2). Model fit is indicated by *R*^2^ and *p*-values. Factors with significant positive (OR > 1) or negative (<1) association are highlighted in bold. For example, significant differences between groups were significantly more often found in journals with impact factor >2.

## Supplementary Materials

The following are available online at www.mdpi.com/1996-1944/9/5/372/s1, Table S1: Included studies; Table S2: Risk of bias.

## Abbreviations

The following abbreviations are used in this manuscript:

FDI	Federation Dentaire International
OR	Odds Ratio
USPHS	United States Public Health Services
