# Supplementary material for: Design and Validity of Randomized Controlled Dental Restorative Trials
Source: Materials (Basel). 2016 May 13;9(5):372. doi: 10.3390/ma9050372 (PMC5503090; doi:10.3390/ma9050372)
Supplement: Supplementary file 1 [file materials-09-00372-s001.pdf]

Supplementary Materials: Design and Validity of Randomized Controlled Dental Restorative Trials

Gerd Göstemeyer, Uwe Blunck, Sebastian Paris and Falk Schwendicke

Table S1. Included studies.

Trial ID	Number of Patients	Lesions per Patient	Number of Lesions	Follow-Up in Months	% Lesions Retained at Follow-Up	Cavity Class	Indication	Dentition	Setting	Super/Infer-Ority Hypothesized	Samplesize Calculation Described	Unit of Randomi-Aation	Analysis Considering Attrition	Trial Registration	Outcome Measure	Significant Differences
Adeleke 2012 [1]	44	7.6	336	12	85.4	V	non-carious	permanent	secondary	no	no	pairwise	not performed	no	USPHS	yes
Ahrari 2010 [2]	36	1.0	36	12	86.1	II	not reported	permanent	secondary	no	no	toothwise	not performed	no	USPHS	no
Alves dos Santos 2010 [3]	48	2.9	141	48	80.9	II	carious	primary	secondary	no	no	pairwise	not performed	no	USPHS	no
Andersson-Wenckert 2006 [4]	61	3.1	190	24	76.8	II	carious	primary	primary	no	no	pairwise	not performed	no	USPHS	no
Aw 2005 [5]	57	3.0	171	36	85.4	V	non-carious	permanent	secondary	no	no	pairwise	not performed	no	USPHS	no
Baracco 2013 [6]	25	3.0	75	24	96.0	I	carious or replacement	permanent	secondary	no	no	pairwise	not performed	no	USPHS	no
Beck 2014 [7]	456	4.0	1805	12	61.4	II	carious or replacement	permanent	secondary	no	no	toothwise	not performed	no	USPHS	no
Blunck 2007 [8]	40	2.0	80	6	95.0	V	non-carious	permanent	secondary	no	no	pairwise	not performed	no	USPHS	no
Boeckler 2012 [9]	50	2.0	100	48	60.0	II	carious or replacement	permanent	secondary	no	no	pairwise	not performed	no	USPHS	no
Bottenberg 2009 [10]	32	4.0	128	60	60.2	II	carious	Permanent	secondary	no	no	toothwise	not performed	no	USPHS	no
Brackett 2007 [11]	50	1.0	50	18	100.0	II	carious or replacement	permanent	secondary	no	no	toothwise	not required	no	USPHS	no
Brackett 2010 [12]	14	5.7	80	24	92.5	V	non-carious	permanent	secondary	no	no	pairwise	not performed	no	USPHS	no
Burgess 2013 [13]	52	3.0	156	24	75.0	V	non-carious	permanent	secondary	yes	no	pairwise	not performed	no	USPHS	no
Burrow 2007 [14]	20	4.6	92	36	59.8	V	non-carious	permanent	secondary	no	no	toothwise	not performed	no	other	yes
Burrow 2012 [15]	11	5.5	60	36	90.0	V	non-carious	permanent	secondary	no	no	pairwise	not performed	no	other	no
Casagrande 2013 [16]	66	2.0	132	18	93.2	II	carious	primary	secondary	no	yes	toothwise	not performed	yes	USPHS	no
Cehreli 2006 [17]	84	2.4	200	24	76.0	I, II	carious	primary	secondary	no	no	pairwise	not performed	no	USPHS	no
Celik 2007 [18]	37	6.8	252	24	59.1	V	non-carious	permanent	secondary	no	no	pairwise	not performed	no	USPHS	yes
Celik 2014 [19]	31	2.6	82	36	75.6	II	carious	permanent	secondary	no	no	pairwise	not performed	no	USPHS	no
Cetin 2013 [20]	54	1.2	67	60	67.2	I	carious or replacement	permanent	secondary	no	no	pairwise	not required	no	USPHS	no

Table S1. *Cont.*

Trial ID	Number of Patients	Lesions per Patient	Number of Lesions	Follow-Up in Months	% Lesions Retained at Follow-Up	Cavity Class	Indication	Dentition	Setting	Super/Infer-Ority Hypothesized	Samplesize Calculation Described	Unit of Randomi-Aation	Analysis Considering Attrition	Trial Registration	Outcome Measure	Significant Differences
Dalton Bittencourt 2005 [21]	25	3.1	78	18	76.9	V	non-carious	permanent	secondary	no	no	pairwise	not performed	no	USPHS	no
Daou 2009 [22]	45	3.3	149	24	62.4	I	carious or replacement	primary	secondary	no	no	toothwise	not performed	no	USPHS	no
Daou 2009 [23]	20	2.0	40	36	60.0	I	carious	primary	secondary	no	no	pairwise	not performed	no	USPHS	no
de Andrade 2014 [24]	41	3.0	123	54	75.6	I	carious or replacement	permanent	secondary	no	yes	pairwise	not performed	no	USPHS	yes
Delbons 2015 [25]	80	1.8	144	18	95.1	I, II	carious or replacement	permanent	primary	no	no	pairwise	not performed	no	USPHS	no
Deliperi 2012 [26]	50	1.5	75	24	61.3	II	carious or replacement	permanent	primary	no	no	toothwise	not performed	no	USPHS	no
Demirci 2008 [27]	32	3.0	96	24	90.6	III	carious	permanent	secondary	no	no	pairwise	not performed	no	USPHS	no
Dall´Orologio 2014 [28]	50	3.0	150	96	53.3	V	non-carious	permanent	secondary	no	yes	pairwise	not performed	no	USPHS	no
Dresch 2006 [29]	37	4.0	148	12	100.0	V	non-carious	permanent	secondary	no	no	pairwise	not required	no	USPHS	no
Dunne 2005 [30]	14	3.6	50	36	94.0	I, II	carious or replacement	permanent	secondary	no	no	toothwise	not performed	no	other	yes
Dutra-Correa 2013 [31]	37	3.2	120	18	76.7	V	non-carious	permanent	secondary	no	no	pairwise	not performed	no	USPHS	no
Efes 2006 [32]	54	2.0	108	24	92.6	I	carious	permanent	secondary	no	no	pairwise	not performed	no	USPHS	no
Efes 2006 [33]	90	1.0	90	24	97.8	I	carious	permanent	secondary	no	no	toothwise	not performed	no	USPHS	no
Efes 2013 [34]	50	2.0	100	24	100.0	I	carious or replacement	permanent	secondary	no	no	pairwise	not required	no	USPHS	no
Eliguzeloglu Dalkilic 2012 [35]	29	8.7	252	24	62.7	V	non-carious	permanent	secondary	no	no	toothwise	not performed	no	USPHS	no
Ergücü 2007 [36]	30	3.2	96	18	93.8	I	carious or replacement	permanent	secondary	no	no	pairwise	not performed	no	USPHS	no
Ermis 2012 [37]	26	6.2	161	24	93.2	V	non-carious	permanent	secondary	no	no	toothwise	not performed	no	other	no
Ernst 2006 [38]	50	2.2	112	24	100.0	II	carious or replacement	permanent	secondary	no	no	pairwise	not required	no	USPHS	no
Ertugrul 2010 [39]	98	2.0	196	12	87.2	II	carious	primary	secondary	no	yes	pairwise	not performed	no	USPHS	no
Fagundes 2009 [40]	33	2.0	66	60	90.9	II	carious or replacement	permanent	secondary	no	no	pairwise	not performed	no	USPHS	no
Frankenberger 2014 [41]	30	2.3	68	96	98.5	II	carious or replacement	permanent	secondary	no	yes	pairwise	not performed	no	USPHS	no
Gallo 2010 [42]	23	2.7	63	36	92.1	I	carious	not reported	secondary	no	yes	toothwise	not performed	no	USPHS	no

Table S1. *Cont.*

Trial ID	Number of Patients	Lesions per Patient	Number of Lesions	Follow-Up in Months	% Lesions Retained at Follow-Up	Cavity Class	Indication	Dentition	Setting	Super/Infer-Ority Hypothesized	Samplesize Calculation Described	Unit of Randomi-Aation	Analysis Considering Attrition	Trial Registration	Outcome Measure	Significant Differences
Gianordoli Neto 2008 [43]	30	2.3	70	12	100.0	II	carious or replacement	permanent	secondary	no	no	pairwise	not required	no	USPHS	no
Goncalves 2013 [44]	33	3.0	100	18	88.0	II	carious or replacement	permanent	secondary	no	yes	toothwise	not performed	no	USPHS	no
Gresnigt 2012 [45]	23	4.2	96	41	87.5	IV	non-carious	permanent	secondary	no	no	pairwise	not required	no	USPHS	no
Häfer 2013 [46]	35	2.6	90	48	98.9	III	carious or replacement	permanent	secondary	no	no	pairwise	not performed	no	USPHS	no
Häfer 2015 [47]	40	2.8	110	36	74.5	V	non-carious	permanent	secondary	no	no	pairwise	not performed	no	other	yes
Jyothi 2011 [48]	32	2.5	80	12	100.0	V	non-carious	permanent	secondary	no	yes	pairwise	not required	no	USPHS	yes
Karaman 2012 [49]	21	6.4	134	24	100.0	V	non-carious	permanent	secondary	yes	no	pairwise	not performed	no	USPHS	no
Kim 2009 [50]	39	3.8	150	24	81.3	V	non-carious	permanent	secondary	no	no	pairwise	not performed	no	USPHS	yes
Koubi 2006 [51]	14	4.0	56	12	100.0	V	non-carious	permanent	secondary	no	no	pairwise	not required	no	USPHS	yes
Krämer 2006 [52]	30	2.4	71	48	66.2	II	not reported	permanent	secondary	no	no	clusterwise	not performed	no	USPHS	yes
Kubo 2006 [53]	8	9.0	72	60	98.6	V	non-carious	permanent	secondary	no	no	pairwise	not performed	no	USPHS	no
Kubo 2009 [54]	23	4.7	108	24	99.1	V	non-carious	permanent	secondary	no	no	pairwise	not performed	no	USPHS	no
Kubo 2010 [55]	22	4.5	98	36	98.0	V	non-carious	permanent	secondary	no	no	pairwise	not performed	no	USPHS	no
Kurokawa 2007 [56]	46	2.1	98	12	100.0	V	non-carious	permanent	secondary	no	no	toothwise	not required	no	USPHS	no
Logueicio 2007 [57]	25	3.1	78	36	76.9	V	non-carious	permanent	secondary	no	yes	pairwise	not performed	no	USPHS	no
Loguercio 2007 [58]	38	3.0	114	12	100.0	III	carious	permanent	secondary	no	no	pairwise	not required	no	USPHS	yes
Mahmoud 2008 [59]	40	3.5	140	24	100.0	I	carious or replacement	permanent	secondary	no	no	pairwise	not performed	no	USPHS	no
Mahmoud 2014 [60]	40	4.0	160	36	100.0	I	carious or replacement	permanent	secondary	no	no	pairwise	not required	no	USPHS	no
Mahmoud 2014 [61]	78	2.0	156	36	100.0	II	carious	permanent	secondary	no	no	pairwise	not performed	no	USPHS	no
Manhart 2010 [62]	43	2.2	96	48	83.3	II	carious or replacement	permanent	secondary	no	no	toothwise	not performed	no	USPHS	no
Monteiro 2010 [63]	26	4.0	105	24	58.1	II	carious	permanent	secondary	no	no	pairwise	not performed	no	USPHS	no
Moosavi 2013 [64]	30	3.0	90	18	91.1	V	non-carious	permanent	secondary	no	no	pairwise	not performed	no	USPHS	no
Moretto 2013 [65]	30	5.8	175	36	89.7	V	non-carious	permanent	secondary	no	no	toothwise	not performed	no	other	no
Pascon 2006 [66]	30	2.3	70	24	85.7	I	carious	primary	secondary	no	no	pairwise	not performed	no	USPHS	yes
Paula 2015 [67]	35	5.1	180	12	100.0	V	non-carious	permanent	secondary	yes	yes	pairwise	not required	no	USPHS and other	no

Table S1. *Cont.*

Trial ID	Number of Patients	Lesions per Patient	Number of Lesions	Follow-Up in Months	% Lesions Retained at Follow-Up	Cavity Class	Indication	Dentition	Setting	Super/Infer-Ority Hypothesized	Samplesize Calculation Described	Unit of Randomi-Aation	Analysis Considering Attrition	Trial Registration	Outcome Measure	Significant Differences
Pazinatto 2012 [68]	33	2.7	90	56	74.4	I	carious or replacement	permanent	secondary	no	no	pairwise	not performed	no	USPHS	no
Perdigao 2005 [69]	35	3.7	128	18	85.9	V	non-carious	permanent	secondary	no	no	toothwise	not performed	no	USPHS	no
Perdigao 2009 [70]	38	3.2	121	24	75.2	II	carious or replacement	permanent	secondary	no	no	pairwise	not performed	no	USPHS	
Perdigao 2012 [71]	39	3.2	125	18	77.6	V	non-carious	permanent	secondary	no	no	pairwise	not performed	no	USPHS	no
Perdigao 2012 [72]	33	2.8	92	12	84.8	V	non-carious	permanent	secondary	no	no	toothwise	not performed	no	USPHS	no
Peumans 2012 [73]	71	2.0	142	156	68.3	V	non-carious	permanent	secondary	no	no	pairwise	not performed	no	other	yes
Pollington 2008 [74]	30	2.0	60	36	100.0	V	non-carious	permanent	secondary	no	no	pairwise	not required	no	USPHS	no
Poon 2005 [75]	65	1.5	98	43	55.1	I	carious	permanent	secondary	no	no	clusterwise	not performed	no	USPHS	no
Qin 2013 [76]	46	2.5	116	24	96.6	V	non-carious	permanent	secondary	no	no	pairwise	not performed	no	USPHS	no
Reis 2009 [77]	30	4.0	120	18	100.0	V	non-carious	permanent	secondary	no	yes	pairwise	not required	no	USPHS	yes
Reis 2009 [78]	84	1.0	84	36	92.9	V	non-carious	permanent	secondary	no	no	toothwise	not performed	no	USPHS	yes
Ritter 2009 [79]	33	3.0	99	96	56.6	V	non-carious	permanent	secondary	yes	yes	toothwise	not performed	no	USPHS	no
Saboia 2006 [80]	14	4.0	56	24	73.2	V	non-carious	permanent	secondary	no	no	pairwise	not performed	no	USPHS	no
Sadeghi 2010 [81]	35	3.0	105	18	100.0	I	carious	permanent	secondary	no	no	pairwise	not required	no	USPHS	no
Santiago 2010 [82]	30	2.3	70	60	78.6	V	non-carious	permanent	secondary	no	no	pairwise	not performed	no	USPHS	yes
Schattenberg 2008 [83]	50	2.1	104	24	100.0	V	carious or non-carious or replacement	permanent	secondary	no	no	pairwise	not performed	no	USPHS	no
Schirrmeister 2009 [84]	43	2.0	86	48	62.8	II	carious	permanent	secondary	no	no	pairwise	not performed	no	USPHS	no
Schmidt 2014 [85]	72	2.2	158	60	67.7	II	carious or replacement	permanent	secondary	no	yes	pairwise	not performed	yes	other	no
Shi 2010 [86]	32	3.1	100	36	80.0	I	carious or replacement	permanent	secondary	no	yes	pairwise	not performed	no	USPHS	no
Söderholm 2013 [87]	21	4.0	84	48	78.6	V	non-carious	permanent	secondary	no	yes	pairwise	not performed	no	USPHS	no
Stojanac 2013 [88]	30	3.0	90	24	100.0	V	non-carious	permanent	secondary	no	no	pairwise	not performed	no	USPHS	no
Torres 2010 [89]	20	2.0	39	36	100.0	II	carious or replacement	permanent	secondary	no	no	toothwise	not performed	no	USPHS	no
Tuncer 2013 [90]	24	5.1	123	24	100.8	V	non-carious	permanent	secondary	no	no	pairwise	not required	no	USPHS	yes
Tuncer 2014 [91]	20	4.9	97	12	100.0	V	non-carious	permanent	secondary	no	no	pairwise	not required	no	USPHS	no
Türkün 2005 [92]	35	4.7	163	12	100.0	V	non-carious	permanent	secondary	no	no	pairwise	not required	no	USPHS	no
Türkün 2008 [93]	24	4.2	100	24	100.0	V	non-carious	permanent	secondary	no	no	pairwise	not performed	no	USPHS	no
Turner 2008 [94]	14	4.0	56	24	96.4	V	non-carious	permanent	secondary	no	no	pairwise	not performed	no	USPHS	no

Table S1. *Cont.*

Trial ID	Number of Patients	Lesions per Patient	Number of Lesions	Follow-Up in Months	% Lesions Retained at Follow-Up	Cavity Class	Indication	Dentition	Setting	Super/Infer-Ority Hypothesized	Samplesize Calculation Described	Unit of Randomi-Aation	Analysis Considering Attrition	Trial Registration	Outcome Measure	Significant Differences
van Dijken 2005 [95]	35	2.1	73	72	91.8	V	non-carious	permanent	secondary	no	no	toothwise	not performed	no	USPHS	yes
van Dijken 2006 [96]	63	1.4	87	72	86.2	II	replacement	permanent	secondary	no	no	toothwise	not performed	no	USPHS	yes
van Dijken 2007 [97]	119	2.8	337	156	81.6	V	non-carious	permanent	secondary	no	no	toothwise	not performed	no	USPHS	yes
van Dijken 2008 [98]	88	3.1	270	156	79.6	V	non-carious	permanent	secondary	no	no	toothwise	not performed	no	USPHS	yes
van Dijken 2009 [99]	50	2.1	106	60	91.5	II	carious or replacement	permanent	secondary	yes	no	pairwise	not performed	no	USPHS	no
van Dijken 2010 [100]	72	1.7	119	96	102.5	V	non-carious	permanent	secondary	no	no	toothwise	not performed	no	USPHS	no
van Dijken 2012 [101]	60	2.3	139	84	97.1	V	non-carious	permanent	secondary	no	no	toothwise	not performed	no	USPHS	no
van Dijken 2013 [102]	67	2.5	169	60	95.3	V	non-carious	permanent	secondary	no	no	pairwise	not performed	no	USPHS	yes
van Dijken 2013 [103]	54	2.1	115	72	96.5	II	carious or replacement	permanent	secondary	no	no	pairwise	not performed	no	USPHS	no
van Dijken 2014 [104]	38	2.8	106	36	98.1	II	carious or replacement	permanent	secondary	no	yes	pairwise	not performed	no	USPHS	no
van Dijken 2014 [105]	78	2.1	165	96	95.8	II	carious or replacement	permanent	secondary	no	no	toothwise	not performed	no	USPHS	no
van Dijken 2014 [106]	52	2.3	122	120	93.4	II	carious or replacement	permanent	secondary	no	no	pairwise	not performed	no	USPHS	no
van Dijken 2015 [107]	82	2.4	196	96	98.0	II	carious or replacement	permanent	secondary	no	yes	pairwise	not performed	no	USPHS	no
van Landuyt 2014 [108]	52	5.1	267	60	89.9	V	non-carious	permanent	secondary	no	no	pairwise	not performed	no	other	no
Yaman 2014 [109]	24	6.0	144	36	83.3	V	non-carious	permanent	secondary	no	no	pairwise	not performed	no	USPHS	yes
Yazici 2014 [110]	28	3.0	84	36	71.4	I	carious	permanent	secondary	no	no	pairwise	not performed	no	USPHS	no
Zander-Grande 2011 [111]	40	4.0	160	24	100.0	V	non-carious	permanent	secondary	no	yes	pairwise	not required	no	USPHS	no
Zander-Grande 2014 [112]	31	4.0	124	24	90.3	V	non-carious	Permanent	secondary	yes	yes	pairwise	performed	no	other	yes
Zhou 2009 [113]	124	2.8	342	12	95.9	V	non-carious	permanent	secondary	no	no	pairwise	not performed	no	USPHS	no
Zulfikaroglu 2008 [114]	51	1.5	75	12	81.3	II	Pulpectomized	primary	secondary	no	no	toothwise	not performed	no	USPHS	yes

Table S2. Risk of bias.

Trial ID	Sequence Generation	Allocation Concealment	Blinding of Operator	Blinding of Examiner	Incomplete Data	Selective Reporting	Overall Risk of Bias
Adeleke 2012 [1]	low	unclear	high	unclear	high	high	unclear/high
Ahrari 2010 [2]	unclear	unclear	high	unclear	low	low	unclear/high
Alves dos Santos 2010 [3]	low	unclear	high	unclear	low	low	unclear/high
Andersson-Wenckert 2006 [4]	unclear	unclear	high	high	low	low	unclear/high
Aw 2005 [5]	unclear	unclear	high	unclear	low	low	unclear/high
Baracco 2013 [6]	unclear	unclear	high	low	low	low	unclear/high
Beck 2014 [7]	low	low	high	unclear	low	low	unclear/high
Blunck 2007 [8]	unclear	unclear	high	unclear	low	low	unclear/high
Boeckler 2012 [9]	unclear	unclear	high	low	low	low	unclear/high
Bottenberg 2009 [10]	low	low	high	low	high	low	unclear/high
Brackett 2007 [11]	unclear	unclear	high	low	high	low	unclear/high
Brackett 2010 [12]	unclear	unclear	high	low	low	low	unclear/high
Burgess 2013 [13]	unclear	unclear	high	low	high	low	unclear/high
Burrow 2007 [14]	low	unclear	high	unclear	low	low	unclear/high
Burrow 2012 [15]	unclear	unclear	high	unclear	low	low	unclear/high
Casagrande 2013 [16]	low	unclear	high	unclear	high	low	unclear/high
Cehreli 2006 [17]	unclear	unclear	high	low	low	low	unclear/high
Celik 2007 [18]	unclear	unclear	high	unclear	high	low	unclear/high
Celik 2014 [19]	low	unclear	high	low	low	low	unclear/high
Cetin 2013 [20]	low	unclear	high	low	low	low	unclear/high
Dalton Bittencourt 2005 [21]	unclear	unclear	high	unclear	low	low	unclear/high
Daou 2009 [22]	unclear	unclear	high	unclear	low	low	unclear/high
Daou 2009 [23]	unclear	unclear	high	high	high	low	unclear/high
de Andrade 2014 [24]	low	low	low	unclear	low	low	unclear/high
Delbons 2015 [25]	unclear	low	high	unclear	low	low	unclear/high
Deliperi 2012 [26]	unclear	unclear	high	low	low	low	unclear/high
Demirci 2008 [27]	unclear	unclear	high	unclear	low	low	unclear/high
Dall´Orologio 2014 [28]	low	low	high	unclear	low	low	unclear/high
Dresch 2006 [29]	low	unclear	high	low	low	low	unclear/high
Dunne 2005 [30]	unclear	high	high	unclear	low	low	unclear/high
Dutra-Correa 2013 [31]	unclear	unclear	high	low	low	low	unclear/high
Efes 2006 [32]	unclear	unclear	high	unclear	low	low	unclear/high
Efes 2006 [33]	unclear	unclear	high	unclear	low	low	unclear/high
Efes 2013 [34]	low	unclear	high	low	low	low	unclear/high
Eliguzeloglu Dalkilic 2012 [35]	unclear	unclear	high	unclear	low	low	unclear/high
Ergücü 2007 [36]	unclear	unclear	high	unclear	low	low	unclear/high
Ermis 2012 [37]	low	unclear	high	low	low	low	unclear/high
Ernst 2006 [38]	unclear	unclear	high	unclear	high	low	unclear/high

Table S2. *Cont.*

Trial ID	Sequence Generation	Allocation Concealment	Blinding of Operator	Blinding of Examiner	Incomplete Data	Selective Reporting	Overall Risk of Bias
Ertugrul 2010 [39]	low	unclear	high	high	high	low	unclear/high
Fagundes 2009 [40]	low	unclear	high	low	low	low	unclear/high
Frankenberger 2014 [41]	unclear	unclear	high	low	low	low	unclear/high
Gallo 2010 [42]	low	unclear	high	unclear	high	low	unclear/high
Gianordoli Neto 2008 [43]	unclear	unclear	high	low	loe	low	unclear/high
Goncalves 2013 [44]	low	unclear	high	low	low	low	unclear/high
Gresnigt 2012 [45]	unclear	unclear	high	low	low	low	unclear/high
Häfer 2013 [46]	unclear	unclear	high	high	low	low	unclear/high
Häfer 2015 [47]	unclear	unclear	high	unclear	low	low	unclear/high
Jyothi 2011 [48]	unclear	unclear	high	low	low	low	unclear/high
Karaman 2012 [49]	unclear	unclear	high	low	low	low	unclear/high
Kim 2009 [50]	unclear	unclear	high	low	low	low	unclear/high
Koubi 2006 [51]	unclear	unclear	high	low	high	high	unclear/high
Krämer 2006 [52]	unclear	unclear	high	unclear	low	low	unclear/high
Kubo 2006 [53]	unclear	unclear	high	low	low	low	unclear/high
Kubo 2009 [54]	unclear	unclear	high	low	low	low	unclear/high
Kubo 2010 [55]	low	unclear	high	low	low	low	unclear/high
Kurokawa 2007 [56]	unclear	unclear	high	unclear	low	low	unclear/high
Logueicio 2007 [57]	low	unclear	high	unclear	low	low	unclear/high
Loguercio 2007 [58]	unclear	unclear	high	low	low	low	unclear/high
Mahmoud 2008 [59]	unclear	unclear	high	unclear	low	low	unclear/high
Mahmoud 2014 [60]	unclear	unclear	high	unclear	low	low	unclear/high
Mahmoud 2014 [61]	low	unclear	high	low	low	low	unclear/high
Manhart 2010 [62]	low	unclear	high	low	low	low	unclear/high
Monteiro 2010 [63]	unclear	unclear	high	high	low	low	unclear/high
Moosavi 2013 [64]	unclear	unclear	high	low	low	low	unclear/high
Moretto 2013 [65]	low	unclear	high	low	low	low	unclear/high
Pascon 2006 [66]	low	unclear	high	low	low	low	unclear/high
Paula 2015 [67]	low	low	high	low	low	low	unclear/high
Pazinatto 2012 [68]	low	unclear	high	low	low	low	unclear/high
Perdigao 2005 [69]	unclear	unclear	high	low	low	low	unclear/high
Perdigao 2009 [70]	unclear	unclear	high	low	low	low	unclear/high
Perdigao 2012 [71]	unclear	unclear	high	low	low	low	unclear/high
Perdigao 2012 [72]	unclear	unclear	high	high	low	low	unclear/high
Peumans 2012 [73]	low	unclear	high	low	high	low	unclear/high
Pollington 2008 [74]	low	unclear	high	high	low	low	unclear/high
Poon 2005 [75]	unclear	unclear	high	unclear	low	low	unclear/high
Qin 2013 [76]	unclear	unclear	high	low	low	low	unclear/high

Table S2. *Cont.*

Trial ID	Sequence Generation	Allocation Concealment	Blinding of Operator	Blinding of Examiner	Incomplete Data	Selective Reporting	Overall Risk of Bias
Reis 2009 [77]	low	unclear	high	high	low	low	unclear/high
Reis 2009 [78]	low	unclear	high	low	low	low	unclear/high
Ritter 2009 [79]	low	unclear	high	unclear	low	low	unclear/high
Saboia 2006 [80]	unclear	unclear	high	low	low	low	unclear/high
Sadeghi 2010 [81]	unclear	unclear	high	low	low	low	unclear/high
Santiago 2010 [82]	unclear	unclear	high	high	low	low	unclear/high
Schattenberg 2008 [83]	unclear	unclear	high	unclear	low	low	unclear/high
Schirrmeister 2009 [84]	low	unclear	high	low	low	low	unclear/high
Schmidt 2014 [85]	low	unclear	high	low	low	low	unclear/high
Shi 2010 [86]	low	unclear	high	low	low	low	unclear/high
Söderholm 2013 [87]	low	unclear	high	unclear	low	low	unclear/high
Stojanac 2013 [88]	unclear	unclear	high	low	low	low	unclear/high
Torres 2010 [89]	unclear	unclear	high	unclear	high	high	unclear/high
Tuncer 2013 [90]	low	low	high	low	low	low	low
Tuncer 2014 [91]	unclear	unclear	high	low	low	low	unclear/high
Türkün 2005 [92]	unclear	unclear	high	unclear	low	low	unclear/high
Türkün 2008 [93]	unclear	unclear	high	unclear	low	low	unclear/high
Turner 2008 [94]	unclear	unclear	high	low	low	low	unclear/high
van Dijken 2005 [95]	unclear	unclear	high	unclear	low	low	unclear/high
van Dijken 2006 [96]	unclear	unclear	high	low	low	low	unclear/high
van Dijken 2007 [97]	unclear	unclear	high	unclear	low	low	unclear/high
van Dijken 2008 [98]	unclear	unclear	high	unclear	low	low	unclear/high
van Dijken 2009 [99]	unclear	unclear	high	unclear	low	low	unclear/high
van Dijken 2010 [100]	unclear	unclear	unclear	unclear	low	low	unclear/high
van Dijken 2012 [101]	low	unclear	high	low	low	low	unclear/high
van Dijken 2013 [102]	unclear	unclear	high	high	low	low	unclear/high
van Dijken 2013 [103]	unclear	unvlear	high	low	low	low	unclear/high
van Dijken 2014 [104]	low	unclear	high	low	low	low	unclear/high
van Dijken 2014 [105]	low	unclear	high	high	low	low	unclear/high
van Dijken 2014 [106]	low	unclear	high	low	low	low	unclear/high
van Dijken 2015 [107]	low	unclear	high	low	low	low	unclear/high
van Landuyt 2014 [108]	low	unclear	high	low	low	low	unclear/high
Yaman 2014 [109]	unclear	unclear	high	low	low	low	unclear/high
Yazici 2014 [110]	low	unclear	high	low	low	low	unclear/high
Zander-Grande 2011 [111]	unclear	unclear	high	low	low	low	unclear/high
Zander-Grande 2014 [112]	low	low	high	low	low	low	low
Zhou 2009 [113]	low	unclear	high	low	low	low	unclear/high
Zulfikaroglu 2008 [114]	unclear	unclear	high	low	low	low	unclear/high

References

1. Adeleke; Oginni, A. Clinical evaluation of resin composite and resin-modified glass ionomer cement in non-carious cervical lesions. *J. West Afr. Coll. Surg.* **2012**, *2*, 21–37.
2. Ahrari, F.; Nojoomian, M.; Moosavi, H. Clinical evaluation of bonded amalgam restorations in endodontically treated premolar teeth: A one-year evaluation. *J. Contemp. Dent. Pract.* **2010**, *11*, 9–16.
3. Alves dos Santos, M.P.; Luiz, R.R.; Maia, L.C. Randomised trial of resin-based restorations in class I and class II beveled preparations in primary molars: 48-month results. *J. Dent.* **2010**, *38*, 451–459.
4. Andersson-Wenckert, I.; Sunnegardh-Gronberg, K. Flowable resin composite as a class II restorative in primary molars: A two-year clinical evaluation. *Acta Odontol. Scand.* **2006**, *64*, 334–340.
5. Aw, T.C.; Lepe, X.; Johnson, G.H.; Mancl, L.A. A three-year clinical evaluation of two-bottle *versus* one-bottle dentin adhesives. *J. Am. Dent. Assoc.* **2005**, *136*, 311–322.
6. Baracco, B.; Perdigao, J.; Cabrera, E.; Ceballos, L. Two-year clinical performance of a low-shrinkage composite in posterior restorations. *Oper. Dent.* **2013**, *38*, 591–600.
7. Beck, F.; Dumitrescu, N.; Konig, F.; Graf, A.; Bauer, P.; Sperr, W.; Moritz, A.; Schedle, A. One-year evaluation of two hybrid composites placed in a randomized-controlled clinical trial. *Dent. Mater.* **2014**, *30*, 824–838.
8. Blunck, U.; Knitter, K.; Jahn, K.R. Six-month clinical evaluation of XP BOND in noncarious cervical lesions. *J. Adhes. Dent.* **2007**, *9* (Suppl. 2), 265–268.
9. Boeckler, A.; Boeckler, L.; Eppendorf, K.; Schaller, H.G.; Gernhardt, C.R. A prospective, randomized clinical trial of a two-step self-etching *vs.* two-step etch-and-rinse adhesive and sem margin analysis: Four-year results. *J. Adhes. Dent.* **2012**, *14*, 585–592.
10. Bottenberg, P.; Jacquet, W.; Alaerts, M.; Keulemans, F. A prospective randomized clinical trial of one bis-GMA-based and two ormocer-based composite restorative systems in class II cavities: Five-year results. *J. Dent.* **2009**, *37*, 198–203.
11. Brackett, W.W.; Browning, W.D.; Brackett, M.G.; Callan, R.S.; Blalock, J.S. Effect of restoration size on the clinical performance of posterior "packable" resin composites over 18 months. *Oper. Dent.* **2007**, *32*, 212–216.
12. Brackett, M.G.; Dib, A.; Franco, G.; Estrada, B.E.; Brackett, W.W. Two-year clinical performance of clearfil SE and clearfil S3 in restoration of unabraded non-carious class V lesions. *Oper. Dent.* **2010**, *35*, 273–278.
13. Burgess, J.O.; Sadid-Zadeh, R.; Cakir, D.; Ramp, L.C. Clinical evaluation of self-etch and total-etch adhesive systems in noncarious cervical lesions: A two-year report. *Oper. Dent.* **2013**, *38*, 477–487.
14. Burrow, M.F.; Tyas, M.J. Clinical evaluation of three adhesive systems for the restoration of non-carious cervical lesions. *Oper. Dent.* **2007**, *32*, 11–15.
15. Burrow, M.F.; Tyas, M.J. Comparison of two all-in-one adhesives bonded to non-carious cervical lesions—Results at 3 years. *Clin. Oral Investig.* **2012**, *16*, 1089–1094.
16. Casagrande, L.; Dalpian, D.M.; Ardenghi, T.M.; Zanatta, F.B.; Balbinot, C.E.; Garcia-Godoy, F.; De Araujo, F.B. Randomized clinical trial of adhesive restorations in primary molars. 18-month results. *Am. J. Dent.* **2013**, *26*, 351–355.
17. Cehreli, Z.C.; Cetinguc, A.; Cengiz, S.B.; Altay, A.N. Clinical performance of pulpotomized primary molars restored with resin-based materials. 24-month results. *Am. J. Dent.* **2006**, *19*, 262–266.
18. Celik, C.; Ozgunaltay, G.; Attar, N. Clinical evaluation of flowable resins in non-carious cervical lesions: Two-year results. *Oper. Dent.* **2007**, *32*, 313–321.
19. Celik, C.; Arhun, N.; Yamanel, K. Clinical evaluation of resin-based composites in posterior restorations: A 3-year study. *Med. Princ. Pract.* **2014**, *23*, 453–459.
20. Cetin, A.R.; Unlu, N.; Cobanoglu, N. A five-year clinical evaluation of direct nanofilled and indirect composite resin restorations in posterior teeth. *Oper. Dent.* **2013**, *38*, E1–E11.
21. Dalton Bittencourt, D.; Ezecelevski, I.G.; Reis, A.; Van Dijken, J.W.; Loguercio, A.D. An 18-months' evaluation of self-etch and etch & rinse adhesive in non-carious cervical lesions. *Acta Odontol. Scand.* **2005**, *63*, 173–178.
22. Daou, M.H.; Tavernier, B.; Meyer, J.M. Two-year clinical evaluation of three restorative materials in primary molars. *J. Clin. Pediatr. Dent.* **2009**, *34*, 53–58.
23. Daou, M.H.; Attin, T.; Gohring, T.N. Clinical success of compomer and amalgam restorations in primary molars. Follow up in 36 months. *Schweiz. Monatsschr. Zahnmed.* **2009**, *119*, 1082–1088.
24. De Andrade, A.K.; Duarte, R.M.; Medeiros e Silva, F.D.; Batista, A.U.; Lima, K.C.; Monteiro, G.Q.; Montes, M.A. Resin composite class I restorations: A 54-month randomized clinical trial. *Oper. Dent.* **2014**, *39*, 588–594.

25. Delbons, F.B.; Perdigao, J.; Araujo, E.; Melo Freire, C.A.; Caldas, D.D.; Cardoso, J.L.; Pagani, M.; Borges, G.A.; Lima, R.B. Randomized clinical trial of four adhesion strategies in posterior restorations-18-month results. *J. Esthet. Restor. Dent.* **2015**, *27*, 107–117.
26. Deliperi, S.; Bardwell, D.N.; Alleman, D. Clinical evaluation of stress-reducing direct composite restorations in structurally compromised molars: A 2-year report. *Oper. Dent.* **2012**, *37*, 109–116.
27. Demirci, M.; Yildiz, E.; Uysal, O. Comparative clinical evaluation of different treatment approaches using a microfilled resin composite and a compomer in class iii cavities: Two-year results. *Oper. Dent.* **2008**, *33*, 7–14.
28. Dall'Orologio, G.D.; Lorenzi, R. Restorations in abrasion/erosion cervical lesions: 8-year results of a triple blind randomized controlled trial. *Am. J. Dent.* **2014**, *27*, 245–250.
29. Dresch, W.; Volpato, S.; Gomes, J.C.; Ribeiro, N.R.; Reis, A.; Loguercio, A.D. Clinical evaluation of a nanofilled composite in posterior teeth: 12-month results. *Oper. Dent.* **2006**, *31*, 409–417.
30. Dunne, S.M.; Abraham, R.; Pankhurst, C.L. A 3-year longitudinal, controlled clinical study of a gallium-based restorative material. *Br. Dent. J.* **2005**, *198*, 355–359.
31. Dutra-Correa, M.; Saraceni, C.H.; Ciaramicoli, M.T.; Kiyan, V.H.; Queiroz, C.S. Effect of chlorhexidine on the 18-month clinical performance of two adhesives. *J. Adhes. Dent.* **2013**, *15*, 287–292.
32. Efes, B.G.; Dorter, C.; Gomec, Y.; Koray, F. Two-year clinical evaluation of ormocer and nanofill composite with and without a flowable liner. *J. Adhes. Dent.* **2006**, *8*, 119–126.
33. Efes, B.G.; Dorter, C.; Gomec, Y. Clinical evaluation of an ormocer, a nanofill composite and a hybrid composite at 2 years. *Am. J. Dent.* **2006**, *19*, 236–240.
34. Efes, B.G.; Yaman, B.C.; Gurbuz, O.; Gumustas, B. Randomized controlled trial of the 2-year clinical performance of a silorane-based resin composite in class 1 posterior restorations. *Am. J. Dent.* **2013**, *26*, 33–38.
35. Eliguzeloglu Dalkilic, E.; Omurlu, H. Two-year clinical evaluation of three adhesive systems in non-carious cervical lesions. *J. Appl. Oral Sci.* **2012**, *20*, 192–199.
36. Ergucu, Z.; Turkun, L.S. Clinical performance of novel resin composites in posterior teeth: 18-month results. *J. Adhes. Dent.* **2007**, *9*, 209–216.
37. Ermis, R.B.; Van Landuyt, K.L.; Cardoso, M.V.; De Munck, J.; Van Meerbeek, B.; Peumans, M. Clinical effectiveness of a one-step self-etch adhesive in non-carious cervical lesions at 2 years. *Clin. Oral Investig.* **2012**, *16*, 889–897.
38. Ernst, C.P.; Brandenbusch, M.; Meyer, G.; Canbek, K.; Gottschalk, F.; Willershausen, B. Two-year clinical performance of a nanofiller vs a fine-particle hybrid resin composite. *Clin. Oral Investig.* **2006**, *10*, 119–125.
39. Ertugrul, F.; Cogulu, D.; Ozdemir, Y.; Ersin, N. Comparison of conventional *versus* colored compomers for class II restorations in primary molars: A 12-month clinical study. *Med. Princ. Pract.* **2010**, *19*, 148–152.
40. Fagundes, T.C.; Barata, T.J.; Carvalho, C.A.; Franco, E.B.; van Dijken, J.W.; Navarro, M.F. Clinical evaluation of two packable posterior composites: A five-year follow-up. *J. Am. Dent. Assoc.* **2009**, *140*, 447–454.
41. Frankenberger, R.; Reinelt, C.; Kramer, N. Nanohybrid *vs.* Fine hybrid composite in extended class II cavities: 8-year results. *Clin. Oral Investig.* **2014**, *18*, 125–137.
42. Gallo, J.R.; Burgess, J.O.; Ripps, A.H.; Walker, R.S.; Maltezos, M.B.; Mercante, D.E.; Davidson, J.M. Three-year clinical evaluation of two flowable composites. *Quintessence Int.* **2010**, *41*, 497–503.
43. Gianordoli Neto, R.; Santiago, S.L.; Mendonca, J.S.; Passos, V.F.; Lauris, J.R.; Navarro, M.F. One year clinical evaluation of two different types of composite resins in posterior teeth. *J. Contemp. Dent. Pract.* **2008**, *9*, 26–33.
44. Goncalves, F.S.; Leal, C.D.; Bueno, A.C.; Freitas, A.B.; Moreira, A.N.; Magalhaes, C.S. A double-blind randomized clinical trial of a silorane-based resin composite in class 2 restorations: 18-month follow-up. *Am. J. Dent.* **2013**, *26*, 93–98.
45. Gresnigt, M.M.; Kalk, W.; Ozcan, M. Randomized controlled split-mouth clinical trial of direct laminate veneers with two micro-hybrid resin composites. *J. Dent.* **2012**, *40*, 766–775.
46. Hafer, M.; Schneider, H.; Rupf, S.; Busch, I.; Fuchss, A.; Merte, I.; Jentsch, H.; Haak, R.; Merte, K. Experimental and clinical evaluation of a self-etching and an etch-and-rinse adhesive system. *J. Adhes. Dent.* **2013**, *15*, 275–286.
47. Hafer, M.; Jentsch, H.; Haak, R.; Schneider, H. A three-year clinical evaluation of a one-step self-etch and a two-step etch-and-rinse adhesive in non-carious cervical lesions. *J. Dent.* **2015**, *43*, 350–361.

48. Jyothi, K.; Annapurna, S.; Kumar, A.S.; Venugopal, P.; Jayashankara, C. Clinical evaluation of giomer- and resin-modified glass ionomer cement in class v noncarious cervical lesions: An *in vivo* study. *J. Conserv. Dent.* **2011**, *14*, 409–413.
49. Karaman, E.; Yazici, A.R.; Ozgunaltay, G.; Dayangac, B. Clinical evaluation of a nanohybrid and a flowable resin composite in non-carious cervical lesions: 24-month results. *J. Adhes. Dent.* **2012**, *14*, 485–492.
50. Kim, S.Y.; Lee, K.W.; Seong, S.R.; Lee, M.A.; Lee, I.B.; Son, H.H.; Kim, H.Y.; Oh, M.H.; Cho, B.H. Two-year clinical effectiveness of adhesives and retention form on resin composite restorations of non-carious cervical lesions. *Oper. Dent.* **2009**, *34*, 507–515.
51. Koubi, S.; Raskin, A.; Bukiet, F.; Pignoly, C.; Toca, E.; Tassery, H. One-year clinical evaluation of two resin composites, two polymerization methods, and a resin-modified glass ionomer in non-carious cervical lesions. *J. Contemp. Dent. Pract.* **2006**, *7*, 42–53.
52. Kramer, N.; Garcia-Godoy, F.; Reinelt, C.; Frankenberger, R. Clinical performance of posterior compomer restorations over 4 years. *Am. J. Dent.* **2006**, *19*, 61–66.
53. Kubo, S.; Kawasaki, K.; Yokota, H.; Hayashi, Y. Five-year clinical evaluation of two adhesive systems in non-carious cervical lesions. *J. Dent.* **2006**, *34*, 97–105.
54. Kubo, S.; Yokota, H.; Yokota, H.; Hayashi, Y. Two-year clinical evaluation of one-step self-etch systems in non-carious cervical lesions. *J. Dent.* **2009**, *37*, 149–155.
55. Kubo, S.; Yokota, H.; Yokota, H.; Hayashi, Y. Three-year clinical evaluation of a flowable and a hybrid resin composite in non-carious cervical lesions. *J. Dent.* **2010**, *38*, 191–200.
56. Kurokawa, H.; Miyazaki, M.; Takamizawa, T.; Rikuta, A.; Tsubota, K.; Uekusa, S. One-year clinical evaluation of five single-step self-etch adhesive systems in non-carious cervical lesions. *Dent. Mater. J.* **2007**, *26*, 14–20.
57. Loguercio, A.D.; Bittencourt, D.D.; Baratieri, L.N.; Reis, A. A 36-month evaluation of self-etch and etch-and-rinse adhesives in noncarious cervical lesions. *J. Am. Dent. Assoc.* **2007**, *138*, 507–514.
58. Loguercio, A.D.; Lorini, E.; Weiss, R.V.; Tori, A.P.; Picinatto, C.C.; Ribeiro, N.R.; Reis, A. A 12-month clinical evaluation of composite resins in class iii restorations. *J. Adhes. Dent.* **2007**, *9*, 57–64.
59. Mahmoud, S.H.; El-Embaby, A.E.; AbdAllah, A.M.; Hamama, H.H. Two-year clinical evaluation of ormocer, nanohybrid and nanofill composite restorative systems in posterior teeth. *J. Adhes. Dent.* **2008**, *10*, 315–322.
60. Mahmoud, S.H.; El-Embaby, A.E.; AbdAllah, A.M. Clinical performance of ormocer, nanofilled, and nanoceramic resin composites in class I and class II restorations: A three-year evaluation. *Oper. Dent.* **2014**, *39*, 32–42.
61. Mahmoud, S.H.; Ali, A.K.; Hegazi, H.A. A three-year prospective randomized study of silorane- and methacrylate-based composite restorative systems in class II restorations. *J. Adhes. Dent.* **2014**, *16*, 285–292.
62. Manhart, J.; Chen, H.Y.; Hickel, R. Clinical evaluation of the posterior composite quixfil in class I and II cavities: 4-year follow-up of a randomized controlled trial. *J. Adhes. Dent.* **2010**, *12*, 237–243.
63. Monteiro, P.M.; Manso, M.C.; Gavinha, S.; Melo, P. Two-year clinical evaluation of packable and nanostructured resin-based composites placed with two techniques. *J. Am. Dent. Assoc.* **2010**, *141*, 319–329.
64. Moosavi, H.; Kimyai, S.; Forghani, M.; Khodadadi, R. The clinical effectiveness of various adhesive systems: An 18-month evaluation. *Oper. Dent.* **2013**, *38*, 134–141.
65. Moretto, S.G.; Russo, E.M.; Carvalho, R.C.; De Munck, J.; Van Landuyt, K.; Peumans, M.; Van Meerbeek, B.; Cardoso, M.V. 3-year clinical effectiveness of one-step adhesives in non-carious cervical lesions. *J. Dent.* **2013**, *41*, 675–682.
66. Pascon, F.M.; Kantovitz, K.R.; Caldo-Teixeira, A.S.; Borges, A.F.; Silva, T.N.; Puppin-Rontani, R.M.; Garcia-Godoy, F. Clinical evaluation of composite and compomer restorations in primary teeth: 24-month results. *J. Dent.* **2006**, *34*, 381–388.
67. Paula, E.A.; Tay, L.Y.; Kose, C.; Mena-Serrano, A.; Reis, A.; Perdigao, J.; Loguercio, A.D. Randomized clinical trial of four adhesion strategies in cervical lesions: 12-month results. *Int. J. Esthet. Dent.* **2015**, *10*, 122–145.
68. Pazinatto, F.B.; Gionordoli Neto, R.; Wang, L.; Mondelli, J.; Mondelli, R.F.; Navarro, M.F. 56-month clinical performance of class I and II resin composite restorations. *J. Appl. Oral Sci.* **2012**, *20*, 323–328.
69. Perdigao, J.; Carmo, A.R.; Geraldeli, S. Eighteen-month clinical evaluation of two dentin adhesives applied on dry *vs* moist dentin. *J. Adhes. Dent.* **2005**, *7*, 253–258.

70. Perdigao, J.; Dutra-Correa, M.; Anauate-Netto, C.; Castilhos, N.; Carmo, A.R.; Lewgoy, H.R.; Amore, R.; Cordeiro, H.J. Two-year clinical evaluation of self-etching adhesives in posterior restorations. *J. Adhes. Dent.* **2009**, *11*, 149–159.
71. Perdigao, J.; Dutra-Correa, M.; Saraceni, C.H.; Ciaramicoli, M.T.; Kiyan, V.H.; Queiroz, C.S. Randomized clinical trial of four adhesion strategies: 18-month results. *Oper. Dent.* **2012**, *37*, 3–11.
72. Perdigao, J.; Dutra-Correa, M.; Saraceni, S.H.; Ciaramicoli, M.T.; Kiyan, V.H. Randomized clinical trial of two resin-modified glass ionomer materials: 1-year results. *Oper. Dent.* **2012**, *37*, 591–601.
73. Peumans, M.; De Munck, J.; Van Landuyt, K.L.; Poitevin, A.; Lambrechts, P.; Van Meerbeek, B. A 13-year clinical evaluation of two three-step etch-and-rinse adhesives in non-carious class-v lesions. *Clin. Oral Investig.* **2012**, *16*, 129–137.
74. Pollington, S.; van Noort, R. A clinical evaluation of a resin composite and a compomer in non-carious class V lesions. A 3-year follow-up. *Am. J. Dent.* **2008**, *21*, 49–52.
75. Poon, E.C.; Smales, R.J.; Yip, K.H. Clinical evaluation of packable and conventional hybrid posterior resin-based composites: Results at 3.5 years. *J. Am. Dent. Assoc.* **2005**, *136*, 1533–1540.
76. Qin, W.; Song, Z.; Ye, Y.Y.; Lin, Z.M. Two-year clinical evaluation of composite resins in non-carious cervical lesions. *Clin. Oral Investig.* **2013**, *17*, 799–804.
77. Reis, A.; Leite, T.M.; Matte, K.; Michels, R.; Amaral, R.C.; Geraldeli, S.; Loguercio, A.D. Improving clinical retention of one-step self-etching adhesive systems with an additional hydrophobic adhesive layer. *J. Am. Dent. Assoc.* **2009**, *140*, 877–885.
78. Reis, A.; Loguercio, A.D. A 36-month clinical evaluation of ethanol/water and acetone-based etch-and-rinse adhesives in non-carious cervical lesions. *Oper. Dent.* **2009**, *34*, 384–391.
79. Ritter, A.V.; Swift, E.J., Jr.; Heymann, H.O.; Sturdevant, J.R.; Wilder, A.D., Jr. An eight-year clinical evaluation of filled and unfilled one-bottle dental adhesives. *J. Am. Dent. Assoc.* **2009**, *140*, 28–37.
80. Saboia Vde, P.; Almeida, P.C.; Rittet, A.V.; Swift, E.J., Jr.; Pimenta, L.A. 2-year clinical evaluation of sodium hypochlorite treatment in the restoration of non-carious cervical lesions: A pilot study. *Oper. Dent.* **2006**, *31*, 530–535.
81. Sadeghi, M.; Lynch, C.D.; Shahamat, N. Eighteen-month clinical evaluation of microhybrid, packable and nanofilled resin composites in class I restorations. *J. Oral Rehabil.* **2010**, *37*, 532–537.
82. Santiago, S.L.; Passos, V.F.; Vieira, A.H.; Navarro, M.F.; Lauris, J.R.; Franco, E.B. Two-year clinical evaluation of resinous restorative systems in non-carious cervical lesions. *Braz. Dent. J.* **2010**, *21*, 229–234.
83. Schattenberg, A.; Werling, U.; Willershausen, B.; Ernst, C.P. Two-year clinical performance of two one-step self-etching adhesives in the restoration of cervical lesions. *Clin. Oral Investig.* **2008**, *12*, 225–232.
84. Schirrmeister, J.F.; Huber, K.; Hellwig, E.; Hahn, P. Four-year evaluation of a resin composite including nanofillers in posterior cavities. *J. Adhes. Dent.* **2009**, *11*, 399–404.
85. Schmidt, M.; Dige, I.; Kirkevang, L.L.; Vaeth, M.; Horsted-Bindslev, P. Five-year evaluation of a low-shrinkage silorane resin composite material: A randomized clinical trial. *Clin. Oral Investig.* **2015**, *19*, 245–251.
86. Shi, L.; Wang, X.; Zhao, Q.; Zhang, Y.; Zhang, L.; Ren, Y.; Chen, Z. Evaluation of packable and conventional hybrid resin composites in class I restorations: Three-year results of a randomized, double-blind and controlled clinical trial. *Oper. Dent.* **2010**, *35*, 11–19.
87. Soderholm, K.J.; Ottenga, M.; Nimmo, S. Four-year clinical evaluation of two self-etching dentin adhesives of different ph values used to restore non-retentive cervical lesions. *Am. J. Dent.* **2013**, *26*, 28–32.
88. Stojanac, I.L.; Premovic, M.T.; Ramic, B.D.; Drobac, M.R.; Stojsin, I.M.; Petrovic, L.M. Noncarious cervical lesions restored with three different tooth-colored materials: Two-year results. *Oper. Dent.* **2013**, *38*, 12–20.
89. Torres, C.R.; Borges, A.B.; Goncalves, S.E.; Pucci, C.R.; de Araujo, M.A.; Barcellos, D.C. Clinical evaluation of two packable resin-based composite restorations: A three-year report. *Gen. Dent.* **2010**, *58*, 338–343.
90. Tuncer, D.; Yazici, A.R.; Ozgunaltay, G.; Dayangac, B. Clinical evaluation of different adhesives used in the restoration of non-carious cervical lesions: 24-month results. *Aust. Dent. J.* **2013**, *58*, 94–100.
91. Tuncer, D.; Celik, C.; Yamanel, K.; Arhun, N. 1 year clinical evaluation of microhybrid composites used in the restoration of non-carious cervical lesions. *Oral Health Dent. Manag.* **2014**, *13*, 366–371.
92. Turkun, L.S. The clinical performance of one- and two-step self-etching adhesive systems at one year. *J. Am. Dent. Assoc.* **2005**, *136*, 656–664.
93. Turkun, L.S.; Celik, E.U. Noncarious class v lesions restored with a polyacid modified resin composite and a nanocomposite: A two-year clinical trial. *J. Adhes. Dent.* **2008**, *10*, 399–405.

94. Turner, E.W.; Shook, L.W.; Ross, J.A.; deRijk, W.; Eason, B.C. Clinical evaluation of a flowable resin composite in non-carious class v lesions: Two-year results. *J. Tenn. Dent. Assoc.* **2008**, *88*, 20–24.
95. Van Dijken, J.W. Retention of a resin-modified glass ionomer adhesive in non-carious cervical lesions. A 6-year follow-up. *J. Dent.* **2005**, *33*, 541–547.
96. Van Dijken, J.W.; Sunnegardh-Gronberg, K. Fiber-reinforced packable resin composites in class II cavities. *J. Dent.* **2006**, *34*, 763–769.
97. Van Dijken, J.W.; Sunnegardh-Gronberg, K.; Lindberg, A. Clinical long-term retention of etch-and-rinse and self-etch adhesive systems in non-carious cervical lesions. A 13 years evaluation. *Dent. Mater.* **2007**, *23*, 1101–1107.
98. Van Dijken, J.W.; Pallesen, U. Long-term dentin retention of etch-and-rinse and self-etch adhesives and a resin-modified glass ionomer cement in non-carious cervical lesions. *Dent. Mater.* **2008**, *24*, 915–922.
99. Van Dijken, J.W.; Lindberg, A. Clinical effectiveness of a low-shrinkage resin composite: A five-year evaluation. *J. Adhes. Dent.* **2009**, *11*, 143–148.
100. Van Dijken, J.W. A prospective 8-year evaluation of a mild two-step self-etching adhesive and a heavily filled two-step etch-and-rinse system in non-carious cervical lesions. *Dent. Mater.* **2010**, *26*, 940–946.
101. Van Dijken, J.W.; Pallesen, U. A 7-year randomized prospective study of a one-step self-etching adhesive in non-carious cervical lesions. The effect of curing modes and restorative material. *J. Dent.* **2012**, *40*, 1060–1067.
102. Van Dijken, J.W. A randomized controlled 5-year prospective study of two hema-free adhesives, a 1-step self etching and a 3-step etch-and-rinse, in non-carious cervical lesions. *Dent. Mater.* **2013**, *29*, e271–e280.
103. Van Dijken, J.W. A 6-year prospective evaluation of a one-step hema-free self-etching adhesive in class II restorations. *Dent. Mater.* **2013**, *29*, 1116–1122.
104. Van Dijken, J.W.; Pallesen, U. A randomized controlled three year evaluation of "bulk-filled"posterior resin restorations based on stress decreasing resin technology. *Dent Mater.* **2014**, *30*, e245–e251.
105. Van Dijken, J.W.; Pallesen, U. Eight-year randomized clinical evaluation of class II nanohybrid resin composite restorations bonded with a one-step self-etch or a two-step etch-and-rinse adhesive. *Clin. Oral Investig.* **2015**, *19*, 1371–1379.
106. Van Dijken, J.W.; Pallesen, U. A randomized 10-year prospective follow-up of class II nanohybrid and conventional hybrid resin composite restorations. *J. Adhes. Dent.* **2014**, *16*, 585–592.
107. Van Dijken, J.W.; Pallesen, U. Randomized 3-year clinical evaluation of class I and II posterior resin restorations placed with a bulk-fill resin composite and a one-step self-etching adhesive. *J. Adhes. Dent.* **2015**, *17*, 81–88.
108. Van Landuyt, K.L.; De Munck, J.; Ermis, R.B.; Peumans, M.; Van Meerbeek, B. Five-year clinical performance of a hema-free one-step self-etch adhesive in noncarious cervical lesions. *Clin. Oral Investig.* **2014**, *18*, 1045–1052.
109. Yaman, B.C.; Dogruer, I.; Gumustas, B.; Efes, B.G. Three-year randomized clinical evaluation of a low-shrinkage silorane-based resin composite in non-carious cervical lesions. *Clin. Oral Investig.* **2014**, *18*, 1071–1079.
110. Yazici, A.R.; Ustunkol, I.; Ozgunaltay, G.; Dayangac, B. Three-year clinical evaluation of different restorative resins in class I restorations. *Oper. Dent.* **2014**, *39*, 248–255.
111. Zander-Grande, C.; Ferreira, S.Q.; da Costa, T.R.; Loguercio, A.D.; Reis, A. Application of etch-and-rinse adhesives on dry and rewet dentin under rubbing action: A 24-month clinical evaluation. *J. Am. Dent. Assoc.* **2011**, *142*, 828–835.
112. Zander-Grande, C.; Amaral, R.C.; Loguercio, A.D.; Barroso, L.P.; Reis, A. Clinical performance of one-step self-etch adhesives applied actively in cervical lesions: 24-month clinical trial. *Oper. Dent.* **2014**, *39*, 228–238.
113. Zhou, Z.; Yu, S.; Jiang, Y.; Lin, Y.; Xiong, Y.; Ni, L. A randomized, controlled clinical trial of one-step self-etching adhesive systems in non-carious cervical lesions. *Am. J. Dent.* **2009**, *22*, 235–240.
114. Zulfikaroglu, B.T.; Atac, A.S.; Cehreli, Z.C. Clinical performance of class II adhesive restorations in pulpectomized primary molars: 12-month results. *J. Dent. Child.* **2008**, *75*, 33–43.
